# Public health round-up

**DOI:** 10.2471/BLT.26.011026

**Published:** 2026-10-01

**Global mpox vaccine stockpile**

A new global stockpile of mpox vaccines was launched on 27 August, creating a long-term mechanism to ensure countries can rapidly access vaccines during outbreaks. Funded by Gavi, the Vaccine Alliance, and coordinated by the International Coordinating Group on Vaccine Provision, the stockpile is available to support outbreak response worldwide. Building on the access and allocation mechanism established during the 2024 mpox public health emergency, the initiative aims to ensure timely and equitable vaccine access. As mpox outbreaks continue globally, with most reported cases occurring in the African Region, the stockpile will help protect vulnerable populations, prevent severe illness and strengthen efforts to contain outbreaks and save lives.

**Figure:**
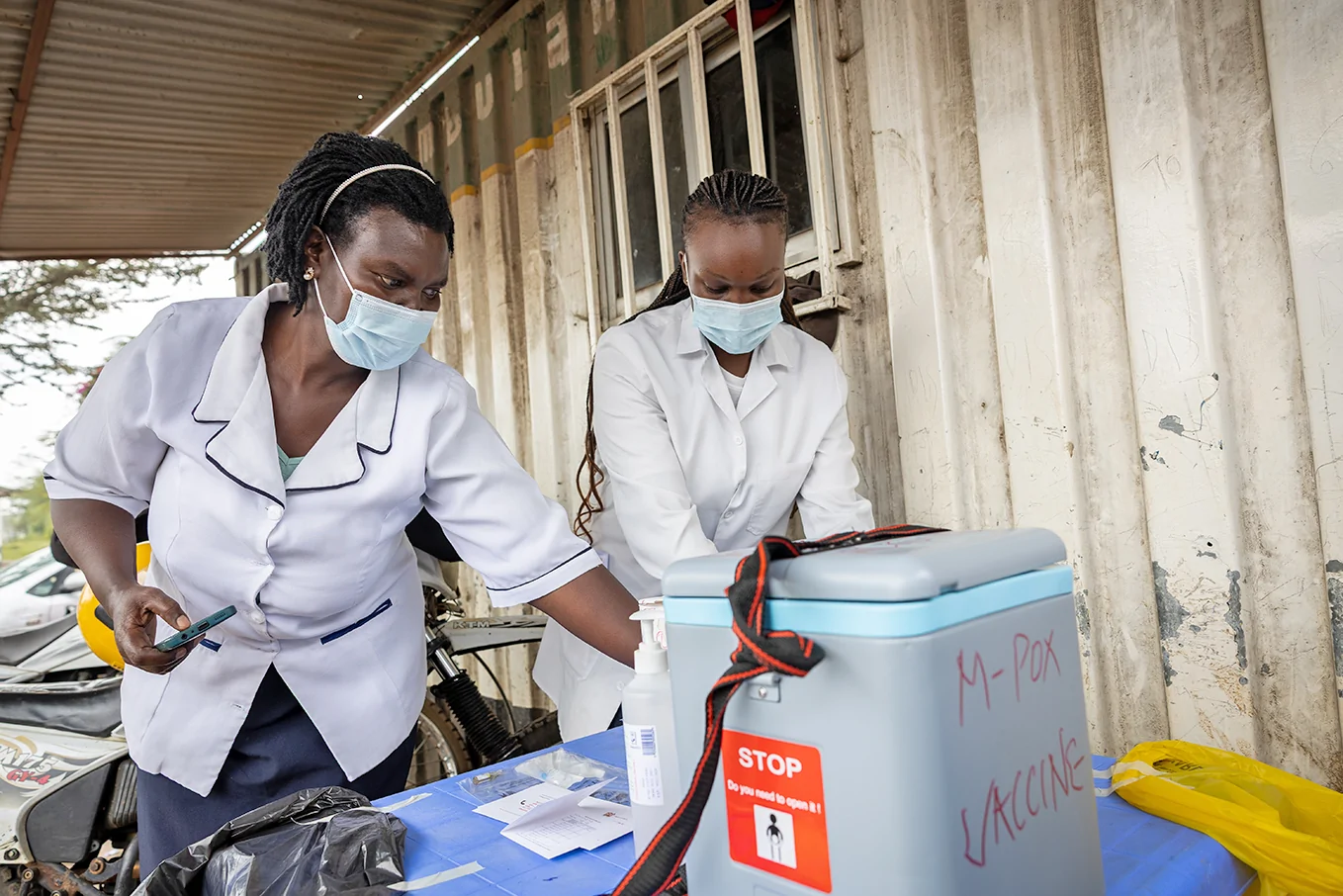
WHO/Ardo Umar Farah

## Primary health care course

The World Health Organization (WHO) has launched a new free online course to help strengthen understanding and implementation of primary health care (PHC). Available through the WHO Academy learning platform, *The primary health care approach: a short course *is designed to support health professionals, policy-makers and other stakeholders in applying PHC principles in practice.

Although PHC has been a global priority for nearly 50 years, its implementation remains uneven and the concept is often misunderstood. The course addresses this gap through five modules covering the importance of PHC, its core components, strategies for strengthening PHC-oriented health systems and approaches to leading health system transformation.

“A shared understanding of what PHC means and how it works can help us realize its full potential and turn this commitment into real improvements in people’s health and in how health systems perform,” said Kalipso Chalkidou, director of WHO’s department of Performance, Financing and Delivery.

https://bit.ly/4xFJdVU

## Data on sexual violence against women

The new technical brief *Measuring non-partner sexual violence against women: data availability, methodological issues and recommendations for good practice* is calling for more consistent and rigorous measurement of non-partner sexual violence against women and girls.

An estimated 263 million women and girls aged 15 years and older, or 8% globally, have experienced sexual violence by someone other than an intimate partner at least once since the age of 15. However, the true scale of the problem is likely higher due to underreporting, stigma and significant gaps in available data.

The brief highlights challenges in comparing data across countries because of differences in survey methods, definitions and populations included. It recommends greater standardization of measurement approaches, the use of behaviourally specific survey questions and stronger safeguards for ethical and confidential data collection.

Improved data are essential to help governments and partners better understand the prevalence of non-partner sexual violence, strengthen prevention and response efforts, support survivors and monitor progress towards ending violence against women and girls.

https://bit.ly/4jfA2HW

## Paediatric cancer medicines prequalification

WHO has issued its first-ever call for manufacturers of childhood cancer medicines to submit products for evaluation through the WHO Prequalification Programme. The initiative aims to expand access to quality-assured medicines for children with cancer, particularly in low- and middle-income countries, where survival rates remain below 30%, compared with more than 80% in many high-income settings.

The expression of interest covers 12 essential childhood cancer medicines, including six priority medicines that require child-friendly formulations and six medicines affected by significant access and supply challenges. The selection was informed by global consultations led through the Global Accelerator for Paediatric Formulations (GAP-f) and the Global Platform for Access to Childhood Cancer Medicines.

By opening a pathway for manufacturers to seek WHO prequalification, the initiative is expected to strengthen the availability of quality-assured medicines for procurement and support countries in improving cancer care for children.

“The expression of interest demonstrates the value of an end-to-end approach to paediatric medicines,” said Martina Penazzato, GAP-f lead, WHO Science for Health department. “Through GAP-f, we have moved systematically from horizon scanning and drug optimization to target product profiles and now to a concrete regulatory pathway that can stimulate manufacturers to develop and submit the products children need.”

https://bit.ly/4ApWIvo

## Eliminating dog-transmitted rabies

WHO has validated Bhutan and Chile for eliminating dog-transmitted rabies as a public health problem, marking important progress in the global effort to end human deaths from dog-mediated rabies by 2030. Bhutan is the first country in the WHO South-East Asia Region to receive the validation, while Chile's validation builds on decades of coordinated action across the Americas, which has made the region a global leader in eliminating dog-transmitted rabies.

Rabies remains one of the world's deadliest neglected tropical diseases and is almost always fatal once symptoms appear. However, human cases can be prevented through mass dog vaccination, effective surveillance and timely access to post-exposure treatment. Globally, dog-transmitted rabies continues to claim tens of thousands of lives each year.

The achievements of Bhutan and Chile reflect years of sustained investment in prevention and control measures. Chile has maintained more than five decades without a human rabies case transmitted by dogs, while Bhutan's success was built on a nationwide One Health approach linking human and animal health services, community engagement and dog vaccination programmes.

The two validations demonstrate that dog-transmitted rabies can be eliminated through strong public health systems, cross-sector collaboration and long-term commitment to prevention and surveillance

https://bit.ly/4xAf61S

https://bit.ly/3SVecPx

## Pandemic agreement negotiations

WHO Member States have advanced negotiations on a proposed pathogen access and benefit-sharing (PABS) system, a key component of the *WHO Pandemic Agreement* designed to support a faster and more equitable response to future pandemics.

The eighth meeting of the Intergovernmental Working Group on the *WHO Pandemic Agreement *brought together governments and stakeholders in Geneva to continue discussions on the PABS annex. The proposed system aims to facilitate the rapid sharing of pathogens with pandemic potential and related sequence information, while ensuring fair and equitable access to vaccines, therapeutics and diagnostics developed from their use.

Finalizing the PABS annex is considered a critical step towards enabling countries to sign and consider ratification of the *WHO Pandemic Agreement*. Once completed, the agreement is expected to strengthen international cooperation, improve preparedness capacities and support a more coordinated response to future global health emergencies. 

https://bit.ly/4xxBCIZ

## New Science Council

WHO has renewed its Science Council, appointing 21 leading scientists and experts from across disciplines, regions and generations to help guide the Organization on emerging scientific developments and innovations with implications for global health.

The council includes specialists in public health, epidemiology, biomedical research, health systems, ageing, engineering, precision medicine and emerging technologies, with representation from all WHO regions as well as two Youth Council members. The inclusion of young scientists through the WHO Youth Council aims to ensure that future generations are represented in discussions on innovations that could shape global health in the decades ahead.

“WHO is strengthening its ability to look ahead, identify transformative developments, and ensure that the benefits of scientific progress are translated into better health for people everywhere,” said WHO Director-General Tedros Adhanom Ghebreyesus. 

By providing independent scientific advice and strategic foresight, the Science Council supports WHO in identifying emerging health risks, opportunities and trends, helping to ensure that advances in science are translated into evidence-based policies and public health action worldwide.

https://bit.ly/4y82Cj6

## Tobacco use and infertility

Tobacco smoking may be reducing the chances of conceiving for millions of people worldwide, according to a new knowledge summary from WHO. The publication highlights growing evidence that tobacco use can affect fertility in both women and men, while exposure to second-hand smoke may also increase reproductive risks. In addition, the publication emphasizes that tobacco use may reduce the success of fertility treatments, including in vitro fertilization, resulting in fewer pregnancies and live births.

WHO recommends that health-care providers routinely inform individuals and couples planning a pregnancy about the reproductive risks of tobacco use and offer support to help them quit. Evidence on newer nicotine and tobacco products is still emerging, but WHO notes that these products may also pose risks to fertility and require further research.

https://bit.ly/4yOPRKm

**Cover photo**

Children queue to receive vaccines during an immunization campaign as part of the diphtheria outbreak response in the Banadir region of Somalia.

**Figure:**
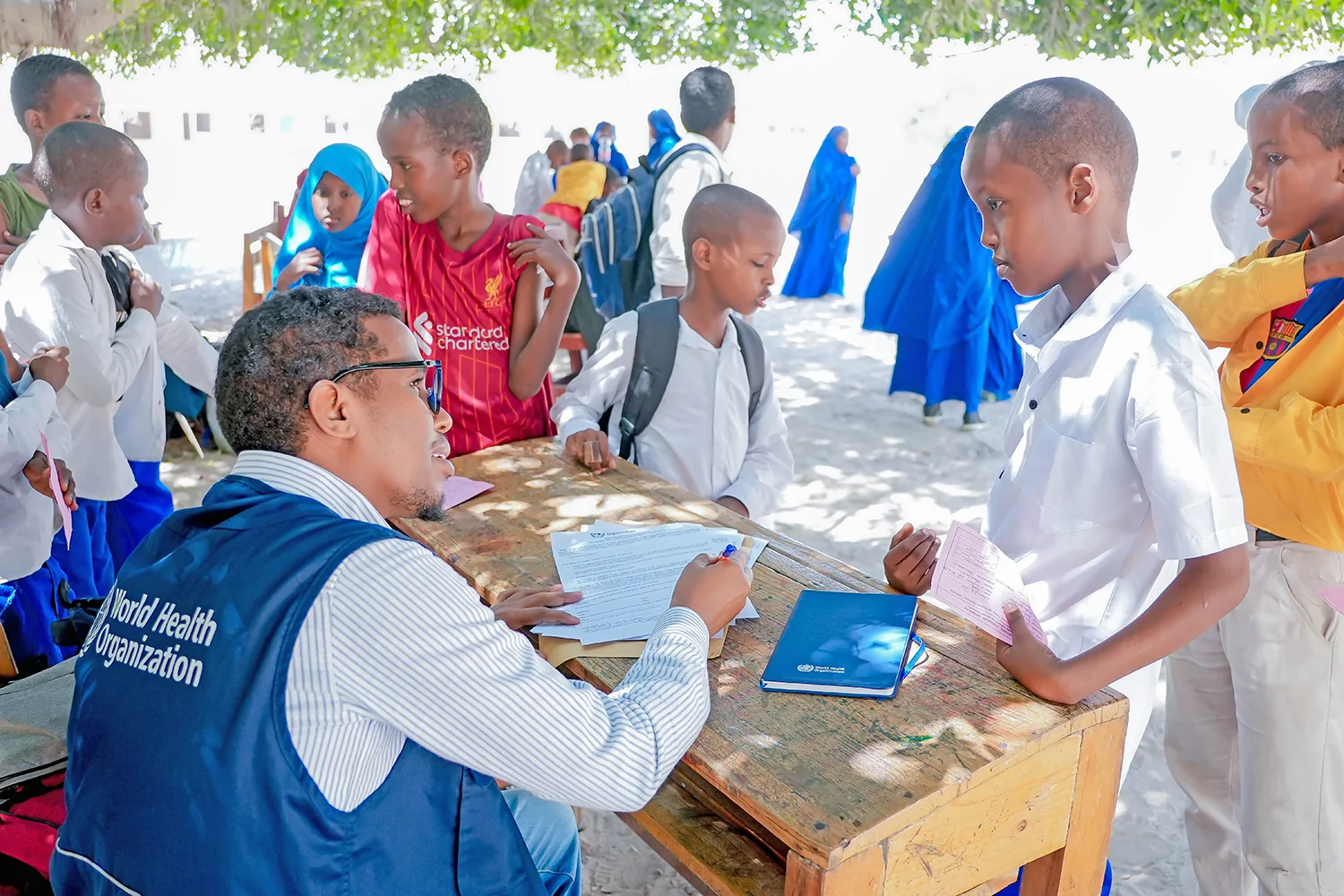
WHO/Abdirahim Caaylaawe

**Looking ahead**

11–13 October. World Health Summit 2026. Berlin, Germany and online. https://bit.ly/3UUbHh1

19–22 October. Forty-ninth Expert Committee on Drug Dependence. Geneva, Switzerland. https://bit.ly/4rkKfVw

28–30 October. Meeting of the Malaria Policy Advisory Group. Online. https://bit.ly/478gQEN

